# Patterns of ambulatory medical care utilization in elderly patients with special reference to chronic diseases and multimorbidity - Results from a claims data based observational study in Germany

**DOI:** 10.1186/1471-2318-11-54

**Published:** 2011-09-13

**Authors:** Hendrik van den Bussche, Gerhard Schön, Tina Kolonko, Heike Hansen, Karl Wegscheider, Gerd Glaeske, Daniela Koller

**Affiliations:** 1 Department of Primary Medical Care, Center of Psychosocial Medicine, University Medical Center Hamburg-Eppendorf, Martinistrasse 52, D-20246 Hamburg, Germany; 2 Department of Medical Biometry and Epidemiology, Center for Experimental Medicine, University Medical Center Hamburg-Eppendorf, Martinistrasse 52, D-20246 Hamburg, Germany; 3 Division of Health Economics, Health Policy and Outcomes Research, Centre for Social Policy Research, University of Bremen, Mary-Somerville-Strasse 5, D-28359 Bremen, Germany

## Abstract

**Background:**

In order to estimate the future demands for health services, the analysis of current utilization patterns of the elderly is crucial. The aim of this study is to analyze ambulatory medical care utilization by elderly patients in relation to age, gender, number of chronic conditions, patterns of multimorbidity, and nursing dependency in Germany.

**Methods:**

Claims data of the year 2004 from 123,224 patients aged 65 years and over which are members of one nationwide operating statutory insurance company in Germany were studied. Multimorbidity was defined as the presence of 3 or more chronic conditions of a list of 46 most prevalent chronic conditions based on ICD 10 diagnoses. Utilization was analyzed by the number of contacts with practices of physicians working in the ambulatory medical care sector and by the number of different physicians contacted for every single chronic condition and their most frequent triadic combinations. Main statistical analyses were multidimensional frequency counts with standard deviations and confidence intervals, and multivariable linear regression analyses.

**Results:**

Multimorbid patients had more than twice as many contacts per year with physicians than those without multimorbidity (36 vs. 16). These contact frequencies were associated with visits to 5.7 different physicians per year in case of multimorbidity vs. 3.5 when multimorbidity was not present. The number of contacts and of physicians contacted increased steadily with the number of chronic conditions. The number of contacts varied between 35 and 54 per year and the number of contacted physicians varied between 5 to 7, depending on the presence of individual chronic diseases and/or their triadic combinations. The influence of gender or age on utilization was small and clinically almost irrelevant. The most important factor influencing physician contact was the presence of nursing dependency due to disability.

**Conclusion:**

In absolute terms, we found a very high rate of utilization of ambulatory medical care by the elderly in Germany, when multimorbidity and especially nursing dependency were present. The extent of utilization by the elderly was related both to the number of chronic conditions and to the individual multimorbidity patterns, but not to gender and almost not to age.

## Background

The increase in the number of elderly people in industrialized countries is generally considered to result in higher health services usage and costs. In Germany, the absolute number of inhabitants 65 years old and more will increase by 41% between 2010 and 2040, and the ratio of elderly people to every 100 persons in the age group 20 to 64 years ("old-age dependency ratio") is even likely to increase by 82% in the same period due to a decreasing birth-death ratio. The number of people 80 years old and older will increase from current 4 million to 10 million by 2050 [1].

It is customary to consider age and concurrent multimorbidity as driving forces in the growing utilization of health services [2-6]. At first glance, the interrelation of old age, morbidity, demand and utilization of health services seems clear. Scientific studies, however, have come to less concordant results. In a review on predictors of health care utilization in the chronically ill, De Boer et al. found ambiguous results with regard to age and gender as predictors of the frequency of physician visits and the degree of hospital utilization [7]. Results presented in the German literature have also been non-conclusive. Bergmann et al. found a curvilinear relationship between age and frequency of physician contact [4], whereas Hessel et al. found no effects of age and gender on the frequency of physician utilization [8]. Laux et al. described an impact on utilization for age but less for gender [9]. Winter et al. saw age as more important than morbidity in explaining ambulatory medical care utilization costs [3], whereas Siegrist declared morbidity patterns to be the major factor responsible for utilization differences between sexes and age-groups [10]. Also, Wiesner et al. described a larger effect of the number of chronic conditions than of age on utilization [11]. As for gender itself, most studies have found a higher utilization pattern in women [12-14], whereas others found no relationship [8]. Coenen et al. also found gender differences in healthcare utilization but stressed the point that such differences were (mostly) related to study design [13].

The manifold inconsistencies in results may be due to differences in the populations studied, study designs, data collection techniques and data analysis methods. For example, many studies on utilization related to age and gender are based on survey data with a recall-biased *a posteriori *investigation. Also, most studies compare utilization patterns in the study population over a long range of age, finding a higher utilization in the elderly as a group but paying less attention to developments within the older age group [3,6]. Finally, the effects of multimorbidity are often not taken into account.

In order to interpret the data on utilization in Germany and the conclusions of this study, a brief introduction to the medical services structure in Germany is provided. Today, health insurance is mandatory for the entire population in Germany, either within a statutory health insurance scheme of the "Bismarckian" type or - for about 10% of the population - a private one. The number of health care professionals - physicians, nurses, physiotherapists etc. - per person is among the highest in Europe [15]. Ambulatory and hospital care are separate sectors within the health care system. As a result, hospital physicians are usually not allowed to work simultaneously in ambulatory care and vice versa. Parallel to the hospital sector, a dense network of ambulatory primary and specialist care exists, mainly provided by physicians in solo or small group offices. These physicians also care for patients in protected living institutions and nursing homes. The group of primary care physicians (PCPs) consists of general practitioners (GPs), all pediatricians and those internists opting to work as a PCP at the moment of opening their practice. The latter account for 60% of all internists. The average density of physicians in 2004 was 1:1,433 population for PCPs and 1:1,218 for specialists [16]. In metropolitan areas the density of specialists is nowadays already much higher than for PCPs.

All physicians function as entre preneurs in a market characterized by free access to all medical disciplines without compulsory gatekeeping. As a result, there are neither rules for the referral to the specialist nor for the re-referral to the GP. In daily life, however, the elderly heavily rely on their PCP, also for referrals. Since 2004, going to see a specialist as the fist physician in a quarter without a referral from the PCP is "punished" by an additional co-payment of 10€.

In order to estimate the future demands for health services, the analysis of current utilization patterns of the elderly and their determinants is crucial. The aim of this study is to analyze ambulatory medical care utilization by patients aged 65 and over in relation to age, gender, number of chronic conditions, individual multimorbidity patterns and nursing dependency in order to determine the influence of these supposedly influential factors.

## Methods

The study is based on an unselected primary care population consisting of all members aged 65 and over (n = 123,224) of a statutory health insurance company operating nationwide in Germany, the Gmünder ErsatzKasse (GEK) in 2004. The GEK insured 1.7 million persons, a figure corresponding to approximately 2.4% of the statutorily insured population. Originally, the GEK primarily insured craftsmen, and the proportion of insured men therefore still exceeds that of women even today. Previous studies have shown that results from the GEK database can be transferred to the German population as a whole if age and gender adjusted [17]. The claims data stem from physicians in the ambulatory medical care sector only. They were provided by GEK in a pseudonymous form. The claims are forwarded on a quarterly basis from the individual physician to the regional physicians' panel association, checked there for comprehensiveness and plausibility and then transferred to the insurances. The observation period of this study was 12 months between January 1 and December 31, 2004.

We selected the most frequent chronic medical conditions in GP surgeries as described in a panel survey ("ADT-Panel") of the Central Research Institute of Statutory Ambulatory Health Care in Germany [18]. Chronicity of conditions was assessed using the "Expert Report for the Selection of 50 to 80 Diseases to be Included in the Morbidity Based Risk Adjustment Scheme" in the German Statutory Health Insurance [19]. In order to capture a comprehensive picture of the disease patterns in individual patients we amended this list for all chronic conditions with a prevalence ≥ 1% in the age group ≥ 65 years in the data set of the Gmünder ErsatzKasse in 2006. The ICD10 codes related to the individual chronic conditions were grouped by an expert panel of family physicians of the Hamburg Institute of Primary Medical Care in order to account for coding variance for the same syndrome among physicians. For example, F00-F03, F05.1, G30, G31 and R54 were grouped under the heading "dementia". The result of this procedure was a list of 46 single codes and code groups further referred to as chronic conditions in this paper. This list includes all frequent somatic and psychological chronic disorders (see additional file 1).

A person was defined as chronically ill if she/he had at least one of the 46 chronic conditions in at least three quarters within the one-year observation period 2004. The three-quarters criterion was chosen in order to avoid transitory or erroneous diagnoses, a usual procedure in using health insurance claims data for research in Germany. Also, acute or sub-acute forms of certain conditions were to be largely excluded by using this criterion. Among the chronically ill, a person was considered as multimorbid when he/she had 3 or more chronic conditions from the list. The criterion of three chronic conditions minimum was considered to be a more valid cut-off score for multimorbidity in elderly patients treated in the ambulatory care setting instead of the usual two chronic conditions criterion, frequently leading to very high rates of multimorbidity in the age group over 65 years [20], as in our data set as well. Recent research supports using the criterion of ≥ 3 conditions for investigations of multimorbidity in the ambulatory care setting, especially when the aim is to compare the multimorbid sample and the non-multimorbid sample (further abbreviated as mm-sample and nmm-sample) in the study population [21,22]. On the basis of this criterion, the total study population of 123,24 persons was divided in a multimorbid sample (abbreviated further as mm-sample) of 76,540 (62%) and a non-multimorbid sample (abbreviated further as nmm-sample) of 46,684 persons (38%). Details on the prevalence of chronic conditions in the study population and the two samples are given in a previous publication [23]. Within the mm-sample, the prevalence of chronic conditions ranged mainly between 3 and 15. We analyzed the effects of the presence of every single chronic condition and of their most frequent triadic combinations ("multimorbidity patterns" [23]). We analyzed the influence of age (available: year of birth), gender, number of chronic conditions (based on the list of 46 described above) and statutory nursing dependency a) on the number of contacts with ambulatory care physician practices per year and b) on the number of different physicians contacted within the year. Statutory nursing dependency is given when a patient receives services from a statutory nursing insurance fund, a parallel agency to the statutory health services insurance scheme. Receiving services from the statutory nursing insurance is used as a proxy for disability in this study, but it should be understood that these figures underestimate the prevalence of disability as disabilities with no or little impact on Activities of Daily Life ("ADL") usually do not lead to receiving benefits from the Insurance.

As for the term "contact", it should be understood that a contact does not necessarily imply an intensive consultation with the physician. Contacts are also needed for prescription renewals or other administrative acts, and in such cases the real contact with the physician may be very short or even absent. This is why we preferred the term contact over terms like visit or consultation.

For every single contact with a physician the database allows to discern if it took place on the basis of a referral document issued by another physician (the primary care physician in the first place) or not.

The main statistical analyses consist of multidimensional frequency tables with the corresponding percentages. Arithmetic means, standard deviations, and confidence intervals were conducted on all continuous outcomes. A linear regression model was performed for the study population (n = 123,224) in order to determine the independent effect of multimorbidity and other factors on physician utilization. We included age, gender, nursing dependency (yes/no), and the number of chronic conditions. Due to the non-linearity of the connection between the frequency of physician contact and the number of chronic conditions, we found an adequate adjustment by adding a non-linear term (logarithm of the number of chronic conditions) to the model.

All analyses were made with the SAS statistical software (Version 9.2) and SPSS 16. Figures were created using R (version 2.12.0) and Windows Microsoft Excel 2003.

The study was approved by the Ethics Committee of the Medical Association of Hamburg (approval no. PV3057).

## Results

### Sociodemographic structure of the study population

The study population consisted of 123,224 patients, 57.6% of which were male and 42.4% were female. Mean age was 72.0 years (71.4 for men and 72.8 for women). People in the mm-sample were older than in the nmm-sample (age mean 72.9 vs. 70.7 years). 60% of the population was multimorbid according to the criterion of three or more chronic conditions. Further details are given in table 1.

**Table 1:** Characteristics of the population under study.

	study population	mm-sample	nmm-sample
Size	100.0%	58.9%	41.1%

Percentage of women	42.4%	44.6%	39.2%

Age (mean, SD)	72.0 (6.1)	72.9 (6.3)	70.7 (5.6)
Female	72.8 (6.7)	73.6 (6.8)	71.4 (6.2)
Male	71.4 (5.7)	72.3 (5.9)	70.2 (5.1)

Mean number of chronic conditions (SD)	3.6 (3.1)	5.7 (2.5)	0.8 (0.8)
Female	3.9 (3.2)	5.7 (2.6)	0.8 (0.9)
Male	3.5 (3.1)	5.6 (2.5)	0.7 (0.8)

Nursing dependency	13.4%	16.9%	8.3%
Female	16.0%	19.6%	10.3%
Male	11.4%	14.8%	7.0%

mm-sample = multimorbid sample; nmm-sample = non-multimorbid sample; SD = standard deviation

The average number of chronic conditions out of the 46-list in the mm-sample was 5.7 (median 5.0) compared to 0.8 in the nmm-sample (median 0). In the nmm-sample, half (51.1%) had no chronic condition from the 46-list and the other half (48.9%) had one or two. Gender differences with regard to the number of chronic conditions in the mm-sample were statistically significant (p < 0.001) but clinically not relevant (5.6 for males vs. 5.7 for females).

### Frequency of contacts with physicians

On average, people aged 65 years and more had 27.9 (SD 23.6) contacts per year with physician practices. Among these, nmm-patients had 15.9 contacts (SD 15.7), whereas mm-patients had more than the double (36.3 contacts; SD 24.5). The latter figure corresponds to 1 contact every 7^th ^working day. As for age, table 2 shows that the number of contacts is slightly higher in the older age groups but the differences between the age groups are relatively small in the mm-sample, especially for women. Differences between men and women regarding contact frequency in general were also remarkably small. In the mm-sample, the overall gender-related difference was 0.6 contacts per year (see table 2 and additional file 2 for details).

**Table 2:** Mean number of contacts/year with physicians according to degree of morbidity, age group and sex among the elderly aged 65 years and more

	non-multimorbid sample					multimorbid sample				
	**mean all**	**SD all**	**male mean**	**female mean**	***p*-value**	**mean all**	**SD all**	**male mean**	**female mean**	***p*-value**

65-69 years	14.6	14.4	13.8	15.9	< 0.001	34.1	23.9	33.4	35.0	< 0.001
70-74 years	16.2	15.7	15.9	16.8	0.004	35.9	24.1	35.9	35.9	0.835
75-79 years	18.2	17.5	18.1	18.3	0.615	38.5	25.0	38.9	37.9	0.009
≥ 80 years	20.6	19.4	20.1	21.0	0.120	39.3	25.4	40.2	38.6	< 0.001
All	15.9	15.7	15.2	17.1	< 0.001	36.3	24.5	36.0	36.6	< 0.001

SD = standard deviation

*p *= statistical significance of difference between women and men.

**Figure 1:**
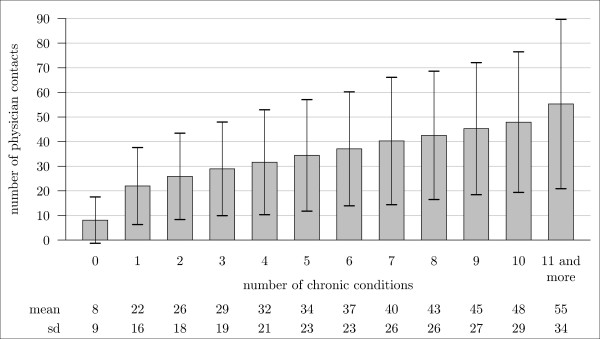
**Mean number of contacts per year with physicians in ambulatory care in relation to the number of chronic conditions among the elderly aged 65 years and over**.

The number of contacts was narrowly related to the number of chronic conditions (see Figure 1 and additional file 2). Persons with none of the 46 chronic conditions under study had on average 8.1 contacts, and this number already increased to 22 (+172%) with the appearance of the first chronic condition. In the mm-sample, the number of contacts rose further from 28.9/year in persons with 3 chronic conditions (= 1 contact every 2 weeks) to 55.3 contacts/year (= > 1 contact/week) for those with 11 chronic conditions and more. The latter number is found for 20% of the mm-sample. All contact number differences according to chronic condition numbers were statistically significant (*p *< 0.001). The high standard deviations point to a great variance in the number of contacts within both samples. Women with a relatively lower number (≤ 6) of chronic conditions had slightly more contacts with physicians than men, whereas men contacted physicians more often than women from 7 chronic conditions on (*p *< 0.001).

The contact frequency also varied with the diagnoses behind the chronic conditions in the triadic multimorbidity patterns of the patients. The average number of contacts was highest for patterns which included anemia (54.3), renal insufficiency (52.1) and urinary incontinence (47.9). The range between the highest and the lowest number of contacts was 20/year, a 59% difference (see additional file 3). A difference of 33% was found for the triadic combinations. Here, the range was between 48.1 contacts/year for hypertension + chronic low back pain + cancer and 36.1 contacts/year equally for the triads hypertension + lipid metabolism disorders + liver disease or obesity (see additional file 4 for results concerning the 50 most frequent triads). The results of the regression analyses on physician utilization are shown in table 3.

**Table 3:** Results of regression analysis on number of physician contacts and number of physicians contacted per year in the study population

	Number of contacts with physicians				Number of physicians contacted			
**R ^2^**	**0.30**				**0.195**			

	**B**	***p-*value**	**95% CI**		**B**	***p-*value**	**95% CI**	

Age (centred)	-0.03	0.0031	-0.05	-0.01	-0.05	<.0001	-0.05	-0.05
Sex (female)	0.05	0.6317	-0.17	0.28	0.16	<.0001	0.12	0.19
Nursing dependency (yes)	10.37	<.0001	10.01	10.72	-0.20	<.0001	-0.25	-0.14
Number of chronic conditions	2.33	<.0001	2.28	2.38	0.24	<.0001	0.23	0.24
Number of chronic conditions (log scale)	1.62	<.0001	157	1.67	0.25	<.0001	0.24	0.26

R 2 = proportion of variance explained by the regression model.

B = beta = regression coefficient

*p *= statistical significance of the effect of the independent variables

For physician contact frequency, the model for the study population explains 30% of the variance in utilization. In accordance with the bivariate analysis, the regression analysis shows that the influence of gender on the number of physician contacts per year was not significant when controlled for other factors. Age was statistically significant (*p *< 0.01) but clinically nearly irrelevant as the frequency of physician contact decreased by only 0.3 contacts for every 10 life years. Controlled for the logarithmic scale of chronic conditions, every chronic condition increased the number of physician visits by 2.3 per year. In particular, chronic conditions leading to services from the statutory nursing insurance increased the number of contacts by 10.4/year. Separate regression analyses for the mm-sample and the nmm-sample did not reveal different results (results not shown in table).

### Number of physicians contacted

On average, patients 65 years old and more contacted 4.8 different physicians (SD 3.3; median: 4). Among them, nmm-patients contacted 3.5 (SD 2.7; median: 3), and mm-patients 5.7 physicians/year (SD 3.3; median 5). 92.6% of the mm-sample and 70.3% of the nmm-sample had one or more contacts with specialists in our study sample. The average number of 5,7 different physicians contacted corresponds to one or more contacts with an average of 4,7 specialists if we assume that every patient had contacts with 1 PCP.

On average, women consulted more physicians than men in the nmm-sample **(**3.3 for men vs. 3.7 for women; p ≤ 0.001), but no statistically significant gender difference was seen in the mm-sample (5.7 for men vs. 5.8 for women, n.s.). With regard to age, the number of physicians contacted decreased with increasing age both in the nmm- and the mm-sample. In women the number of specialists contacted was lower in the older old compared to the younger old whereas this was not the case in men. As a result, women contacted slightly more physicians than men in the age group under 75 but the inverse was found in the age group 75 years and more (see table 4). Further analysis revealed that this decline is mainly due to the fact that multimorbid women saw a gynecologist less frequently after the age of 65 whereas the proportion of multimorbid men seeing a urologist was largely age-group independent.

**Table 4:** Mean number of different physicians contacted per year in the population aged 65 years and more according to degree of morbidity and sex

	non-multimorbid sample				multimorbid sample			
**age groups (years)**	**all mean (SD)**	**male mean (SD)**	**female mean (SD)**	***p *value**	**all mean (SD)**	**male mean (SD)**	**female mean (SD)**	***p *value**

65-69	3.5 (2.8)	3.2 (2.6)	3.9 (3.0)	p ≤ 0.001	6.0 (3.4)	5.7 (3.2)	6.4 (3.6)	p ≤ 0.001
70-74	3.5 (2.7)	3.4 (2.7)	3.6 (2.8)	p ≤ 0.001	5.8 (3.3)	5.8 (3.2)	5.9 (3.5)	p ≤ 0.001
75-79	3.4 (2.6)	3.5 (2.6)	3.4 (2.7)	p = 0.80	5.7 (3.3)	5.9 (3.3)	5.5 (3.3)	p ≤ 0.001
≥ 80	3.2 (2.5)	3.4 (2.7)	3.1 (2.3)	p ≤ 0.001	5.0 (3.1)	5.5 (3.2)	4.7 (2.9)	p ≤ 0.001
All	3.5 (2.7)	3.3 (2.6)	3.7 (2.8)	p ≤ 0.001	5.7 (3.3)	5.7 (3.2)	5.8 (3.4)	p ≤ 0.001

SD = standard deviation

*p *= statistical significance of difference between means in women and men.

**Figure 2:**
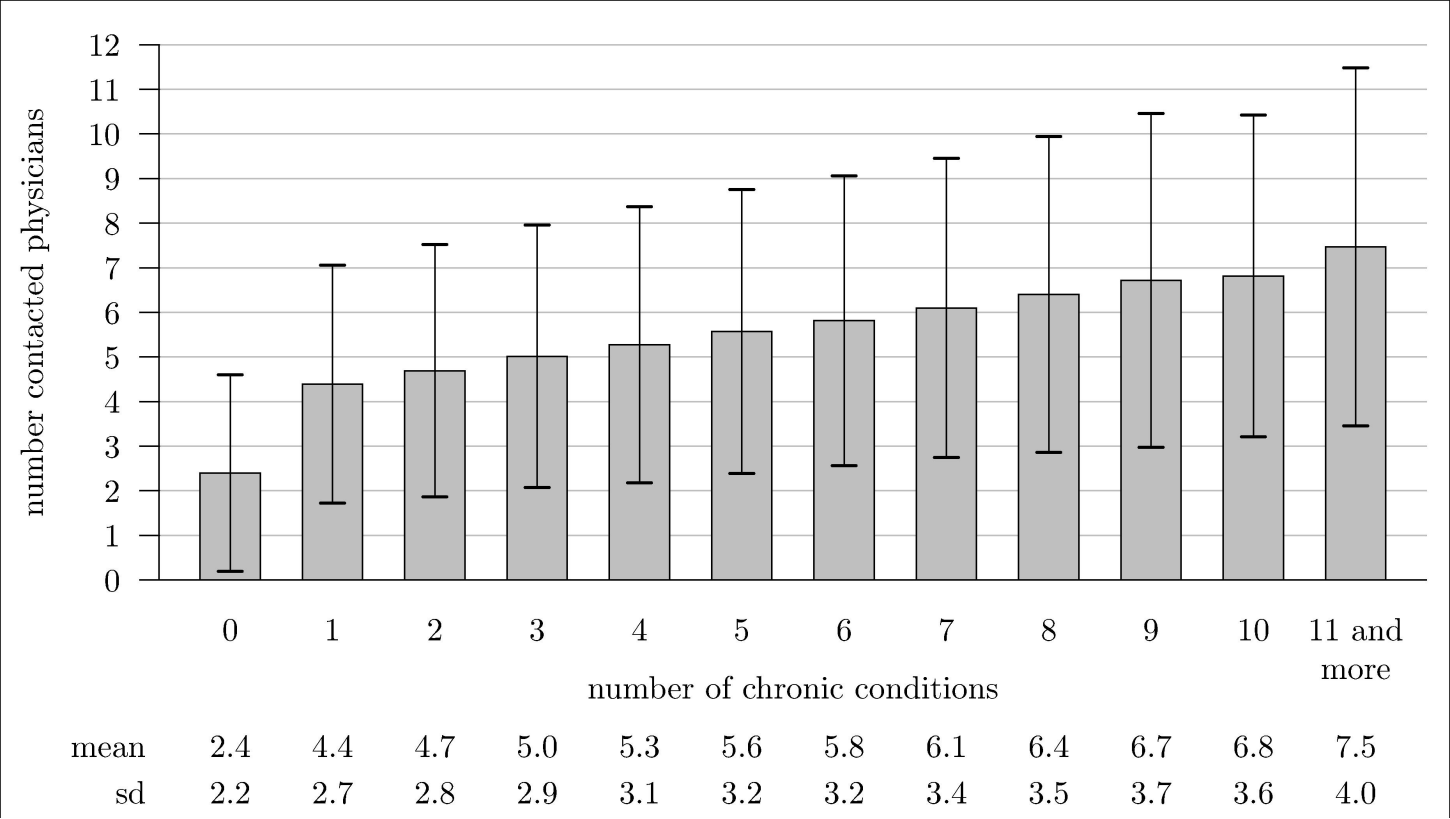
**Mean number of different physicians in ambulatory care contacted per year in relation to the number of chronic conditions in the elderly aged 65 years and over**.

As expected, he number of contacted physicians was also related to the number of chronic conditions. However, the largest increase in the number of physicians contacted (from 2.4 to 4.4) took place between 0 and 1 chronic condition, whereas the increase was less than 0.5 physician/year for every further chronic condition (see Figure 2).

The number of physicians contacted also varied with individual chronic conditions. This number was lowest for patterns including dementia (5.2) and cardiac insufficiency (5.3), and highest for patterns including cancer (7.0) and sexual dysfunction (7.1). It is also striking that patterns including anxiety (6.7) and somatoform disorders (6.8) belong to the group of conditions leading to contacts with many physicians (see additional file 5 for details). The range between the means of the number of physicians contacted (5.2 vs. 7.1) was 1.9, and the range for the medians 1 physician (except for patterns including dementia). This relatively small difference in numbers of physicians contacted for patterns including specific chronic conditions was also seen in the triadic combinations. Here, the range was between a maximum of 7.2 physicians/year (SD 3.7) for patterns which included the triad hypertension + chronic low back pain + severe vision reduction and a minimum of 5.4 physicians per year equally for the patterns including triads hypertension + lipid metabolism disorders + obesity or purine/pyrimidine metabolism disorders/gout.

The results of the regression on the number of physicians contacted (see table 3) show a statistically significant effect of every variable when controlling for all other factors. Still, the clinical relevance was rather low: For age, an increase by 10 life years corresponds to a decrease of utilization of 0.5 physicians per year, and women contacted 0.16 more physicians in comparison to men in the mm-sample. As in the bivariate analysis, four chronic conditions more are needed to increase the number of contacted physicians by 1. The model explains 20% of the variance, which means that several other factors play an important role. Regression analysis for the mm-sample and the nmm-sample did not reveal different results (results not shown in table).

## Discussion

The aim of this study was to analyze ambulatory medical care utilization by patients aged 65 and over in relation to chronic diseases and multimorbidity in Germany. We found a high rate of utilization of ambulatory medical care services by the elderly. Being multimorbid corresponded to more than the double of contacts compared to the non-multimorbid sample. On average, multimorbid elderly persons had 36 contacts with physician practices per year. Some 20% of the multimorbid elderly had one contact with a physician practice every week. In general, both age differences among the elderly and sex had at best a small influence on the number of contacts with physicians and the number of contacted medical professionals in the mm-sample. The number of contacts per year varied largely according to individual chronic conditions and to their combinations in the multimorbidity patterns. Chronic conditions leading to nursing dependency was the most important factor related to high utilization rates.

It is customary to pretend an almost inevitable increase of utilization of medical services due to the growing number of elderly people in the population worldwide. In our study, however, the bivariate analysis showed only a very moderate increase in the number of contacts (15%) between the youngest and the oldest age group in the mm-sample, and this effect disappeared when controlled for other factors. The number of physicians contacted even decreased among the oldest old, especially in females. These results contradict the thesis on the association of growing old and increasing use of medical services. Multiple reasons may explain this non-association. It may be that people become older without a parallel increase of the number of chronic diseases and/or of disease burden and/or complications, e.g. due to earlier diagnosis, success of treatment and/or secondary prevention. This hypothesis is known as compression of morbidity, a thesis still under discussion [24]. There is still no scientific proof that an increasing longevity is associated with a shortened period of morbidity and/or disability, but many studies point in this direction [25]. In our study, the difference in the number of chronic conditions between the youngest and the oldest age group was only 1, although the difference in average age between the youngest and the oldest age group was 17 years [23]. The demand for services by the elderly also depends on the social setting (socially integrated or living alone) or the living conditions of the patients (in the community or in the nursing home). Also, explicit rationing and/or silent age discrimination by professionals might contribute to the cessation of the increase of utilization in the oldest old. On the other hand, the absence of an age-related increase in utilization may also be due to the one-year time span of this study, since frequent utilizers may die at an earlier age. Here, analyses of utilization covering several years are needed. As for research on utilization by patients and for care supply planning, the potential survival phenomenon described above is irrelevant as the object of health services research is the real population under actual care conditions.

In contrast to many studies [12,13,26,27], we also did not find clear-cut signs of higher utilization patterns in the multimorbid female elderly compared to men. In the bivariate analysis, the difference in visit numbers was small for all age groups, whereas the number of different physicians contacted was higher for multimorbid women in the younger age groups but lower in the older groups. Our data suggest that the number of specialists seen by multimorbid patients decreases with age in female, but not in male patients, except for the oldest old. Of course, a study covering a 12 months time span cannot definitively prove the asserted non-associations, for which reason observations over several years will follow. The small effect of gender was confirmed in the regression analysis.

The positive association between the number of chronic conditions and the two utilization indicators in this study was not linear. Instead, both indicators showed the relatively largest increase from 0 to 1 chronic condition. In other words, the increase in utilization started already with the appearance of the first chronic condition and grows relatively consistently with every further number. The number of chronic conditions had a great influence on utilization in bivariate analyses, but its effect also became unspectacular when controlling for other factors as every additional chronic condition increased the number of contacts by 2.3 per year only. The only really important factor for high utilization was ADL-related disability, as expressed by the reception of services from the statutory nursing insurance system. Nursing dependency led to a small decrease in the number of physicians contacted but to 10 more visits to physicians per year. This association underscores the idea that primary and/or secondary prevention of disability might lead to a reduction of the utilization of ambulatory medical services.

The increase in utilization of ambulatory medical care due to chronic diseases and mutlimorbidity found in this study confirms the results of many other studies. In a comprehensive review of the literature by Gijsen et al., the authors concluded that "comorbidity was consistently related to health care utilization (costs, length of hospital stay, and number of physician visits)" [28]. Also, the German Robert-Koch-Institute study based on survey methods found a doubling of the number of physician contacts in ambulatory care within the multimorbid population under 75 years in Germany [11].

The number of contacts and of contacted physicians also varied with the presence of individual chronic conditions and of their (triadic) combinations in the multimorbidity patterns of the patients. The number of contacts varied between 35 and 54 per year and the number of contacted physicians between 5 and 7 depending on the presence of individual chronic conditions. Similar variances are also found for patterns which included the 50 most frequent triadic combinations (maximum 48 and minimum 36 contacts). Further research is needed to explain these variances. At first glance, the number of contacts is not related to the question if the diagnoses are related to one or several specialist disciplines ("morbidity mix"). Neither is there an obvious association between the number of contacts and the number of contacted physicians. In any case, the data suggest that the utilization figures are independent of the prevalence of the chronic conditions and their combinations.

With an average of 36/year, the number of contacts of multimorbid elderly with physicians in ambulatory care is likely to be far greater in Germany than in the health care systems of other countries. In the Netherlands, the number of contacts per year with physicians in ambulatory care is around 11 in patients with chronic disease(s), 5 of them with specialists [29]. Nie et al. reported 10 visits to ambulatory care physicians in Ontario in 2005/06 [6]. For the United States of America, Starfield reported 15.5 visits (6.6 visits to the PCP and 9.0 visits to specialists) in the sample with the highest multimorbidity burden in a Medicare sample aged 65 years and older in 1999 [30]. Also, the average number of contacted specialists (estimated 4,7 for Germany) and the proportion of the multimorbid population referred to specialists (93%) is obviously higher than in other countries. Forrest et al. found that 14% of the population in the United Kingdom and 30 to 37% in managed care plans in the United States of America were referred to specialists in 2002 [31]. The percentage of elderly with at least one chronic condition referred to at least one specialist was 80% in the Netherlands in 2004 [29]. Nie found 3 visits to specialists per year for the elderly in Ontario, Canada [6]. Regardless of the comparison problems of health systems in general and the definition of contact or visit in particular as described in the methods section, Germany seems to be leading country in the world with regard to the proportion of people referred to specialists, the number of different specialists visited and the number of contacts with both PCPs and specialists per year. Further research is needed to explain these exceptional features of the German ambulatory health care system. Apart from patient-based utilization habits, the contribution of morbidity-independent bureaucratic regulations in the insurance system (e.g. prescription regulations, budget limits, co-payment rules etc.) deserve more attention with regard to their effects on utilization. Like in the United States of America, specialists in Germany play an important role in caring for common conditions, not particularly when the level of comorbidity is high or the individual chronic condition warrants highly specialized care [30,32]. As many consultations of specialists end with recommendations for further diagnostics and/or drug prescriptions, problems of guidance, documentation, coordination and cooperation between professionals arise, for which tools and routines are largely lacking.

Our study has weaknesses but also strengths. As this study is based on claims data, diagnoses were not clinically verified by professionals specially trained for this study. Erroneous and transitory diagnoses were minimized by monitoring the persons over a whole year, only acknowledging a condition as chronic if a ICD code from the 46er list was found in at least three quarters of the year. Privately insured patients (some 10% of the population) were not included in this study. The claims data do not allow the analysis of other important aspects of utilization, such as subjective utilization needs, disease severity or socio-economic characteristics of patients. On the other hand, insurance claims data do allow the analysis of large populations over long periods of time, including those living in protected institutions and in nursing homes and those of frail individuals as well as of the oldest of the elderly, all of whom are frequently excluded from field studies. The same applies to the lack of selection bias with regard to the service providers, an even greater problem in field research. Also, recall bias and social desirability problems in interviews concerning the utilization of services are excluded.

In this study we only examined the utilization of ambulatory physician services but we intend to expand this analysis to hospital utilization, physiotherapy services, and pharmaceutical use in order to obtain a complete picture on utilization and cost of medical care for the elderly in Germany.

## Conclusions

In absolute terms, we found a very high rate of utilization of ambulatory medical care by the elderly in Germany, when multimorbidity and especially nursing dependency were present. This extent of utilization was related to the number of chronic conditions and to the presence of individual chronic conditions or their combinations in the multimorbidity pattern of the patients. On the other hand, the extent of utilization was not related to gender and was almost not higher in the older age groups among the elderly. These figures exceed those published by other countries by far. Further research is needed to explain these exceptional features of the German ambulatory health care system.

## Competing interests

GG received funding from statutory health insurance companies for scientific analyses, among them from the GEK.

## Authors' contributions

HvdB, KW, GS and DK conceived the study. GG acquired the data. TK, HH, DK and GS performed the data analysis and contributed to their interpretation. HH coordinated the project. HvdB drafted the manuscript. All authors revised the manuscript critically and gave their approval for publication.

## Pre-publication history

The pre-publication history for this paper can be accessed here:

http://www.biomedcentral.com/1471-2318/11/54/prepub

## Supplementary Material

Additional file 1 **List of 46 diagnoses of chronic conditions and corresponding ICD codes used in this study (PDF)**. Click here for file

Additional file 2 **Mean number of contacts per year with physicians in ambulatory care in the elderly aged 65 and over according to degree of morbidity and sex (PDF)**. Click here for file

Additional file 3 **Mean number of contacts per year with physicians in ambulatory care in the elderly aged 65 and over according to individual chronic conditions in the study population (PDF)**. Click here for file

Additional file 4 **Mean number of contacts per year with physicians in ambulatory care in the multimorbid elderly aged 65 and more according to triadic combinations of chronic conditions ranked according to number of contacts (PDF)**. Click here for file

Additional file 5 **Mean number of physicians contacted per year in ambulatory care in the elderly population according to individual chronic conditions (PDF)**. Click here for file
