# Supplementary material for: Patterns of ambulatory medical care utilization in elderly patients with special reference to chronic diseases and multimorbidity - Results from a claims data based observational study in Germany
Source: BMC Geriatr. 2011 Sep 13;11:54. doi: 10.1186/1471-2318-11-54 (PMC3180370; doi:10.1186/1471-2318-11-54)
Supplement: Additional file 1 — List of 46 diagnoses of chronic conditions and corresponding ICD codes used in this study (PDF). [file 1471-2318-11-54-S1.PDF]

Additional file 1: List of the 46 chronic conditions and the corresponding ICD codes used in this study

No	Chronic condition	ICD Codes
1	Hypertension	I10-I15
2	Lipid metabolism disorders	E78
3	Chronic low back pain	M40-M45, M47, M48.0-M48.2, M48.5-M48.9 M50-M54
4	Severe vision reduction	H17-H18, H25-H28, H31, H33, H34.1-H34.2, H34.8-H34.9, H35-H36, H40, H43, H47, H54
5	Joint arthrosis	M15-M19
6	Diabetes mellitus	E10-E14
7	Chronic ischemic heart disease	I20, I25, I21
8	Thyroid diseases	E01-E05, E06.1-E06.3, E06.5, E06.9, E07
9	Cardiac arrhythmias	I44-I45, I46.0, I46.9, I47-I48, I49.1-I49.9
10	Obesity	E66
11	Purine/pyrimidine metabolism disorders/gout	E79, M10
12	Prostatic hyperplasia	N40
13	Lower limb varicosis	I83, I87.2
14	Liver disease	K70, K71.3-K71.5, K71.7, K72.1, K72.7, K72.9, K73-K74, K76
15	Depression	F32-F33
16	Asthma/COPD	J40-J45, J47
17	Noninflammatory gynaecological problems	N81, N84-N90, N93, N95
18	Atherosclerosis/PAOD	I65-I66, I67.2, I70, I73.9
19	Osteoporosis	M80-M82
20	Renal insufficiency	N18-N19
21	Cerebral ischemia/chronic stroke	I60-I64, I69, G45
22	Cardiac insufficiency	I50
23	Severe hearing loss	H90, H91.0, H91.1, H91.3, H91.8, H91.9
24	Chronic cholecystitis/Gallstones	K80, K81.1
25	Somatoform disorders	F45
26	Hemorrhoids	I84
27	Intestinal diverticulosis	K57
28	Rheumatoid arthritis/Chronic polyarthritis	M05-M06, M79.0
29	Cardiac valve disorders	I34-I37
30	Neuropathies	G50-G64
31	Dizziness	H81-H82, R42
32	Dementia	F00-F03, F05.1, G30, G31, R54
33	Urinary incontinence	N39.3-N39.4, R32
34	Urinary tract calculi	N20
35	Anaemia	D50-D53, D55-D58, D59.0-D59.2, D59.4-D59.9, D60.0, D60.8, D60.9, D61, D63-D64
36	Anxiety	F40-F41
37	Psoriasis	L40
38	Migraine/chronic headache	G43, G44
39	Parkinson’s disease	G20-G22
40	Cancer	C00-C14, C15-C26, C30-C39, C40-C41, C43-C44, C45-C49, C50, C51-C58, C60-C63, C64-C68, C69-C72, C73-C75, C81-C96, C76-C80, C97, D00-D09, D37-D48
41	Allergy	H01.1, J30, L23, L27.2, L56.4, K52.2, K90.0, T78.1, T78.4, T88.7
42	Chronic gastritis/GERD	K21, K25.4-K25.9 K26.4-K26.9 K27.4-K27.9 K28.4-K28.9 K29.2-K29.9
43	Sexual dysfunction	F52, N48.4
44	Insomnia	G47, F51
45	Tobacco abuse	F17
46	Hypotension	I95
