# Supplementary material for: Patterns of ambulatory medical care utilization in elderly patients with special reference to chronic diseases and multimorbidity - Results from a claims data based observational study in Germany
Source: BMC Geriatr. 2011 Sep 13;11:54. doi: 10.1186/1471-2318-11-54 (PMC3180370; doi:10.1186/1471-2318-11-54)
Supplement: Additional file 2 — Mean number of contacts per year with physicians in ambulatory care in the elderly aged 65 and over according to degree of morbidity and sex (PDF). [file 1471-2318-11-54-S2.PDF]

Additional file 2: Frequency of contact with physicians in ambulatory care in the elderly aged 65 and over according to degree of morbidity and gender

Number of chronic conditions	Total sample			men		women	
	number	mean contacts/year	SD	mean contacts/year	SD	mean contacts/year	SD
0	25888	8.1	9.4	7.8	9.1	8.6	9.9
1	11134	22.0	15.6	21.4	15.4	22.7	15.9
2	13654	25.9	17.5	25.6	17.8	26.3	17.2
All nmm	*50676*	*15.9*	*15.7*	*15.2*	*15.5*	*17.1*	*16.1*
3	14765	28.9	19.0	28.6	19.4	29.3	18.6
4	14211	31.6	21.3	31.3	22.0	32.0	20.3
5	12393	34.4	22.7	34.2	22.5	34.7	22.8
6	9404	37.1	23.1	36.8	24.3	37.4	21.6
7	7133	40.3	25.9	40.7	27.7	39.7	23.4
8	5136	42.5	26.1	43.0	27.2	41.9	24.7
9	3481	45.3	26.8	45.8	27.1	44.7	26.5
10	2357	47.9	28.6	48.0	30.0	47.8	26.9
11 and more	3668	55.3	34.4	56.0	36.1	54.5	32.5
All mm	*72548*	*36.3*	*24.5*	*36.0*	*25.2*	*36.6*	*23.7*
All	123224	27.9	23.6	27.0	23.9	29.1	23.1

nmm = non-mulimorbid sample
mm = mulimorbid sample
