# Supplementary material for: Patterns of ambulatory medical care utilization in elderly patients with special reference to chronic diseases and multimorbidity - Results from a claims data based observational study in Germany
Source: BMC Geriatr. 2011 Sep 13;11:54. doi: 10.1186/1471-2318-11-54 (PMC3180370; doi:10.1186/1471-2318-11-54)
Supplement: Additional file 3 — Mean number of contacts per year with physicians in ambulatory care in the elderly aged 65 and over according to individual chronic conditions in the study population (PDF). [file 1471-2318-11-54-S3.PDF]

Additional file 3: Mean number of contacts per year with physicians in ambulatory care in the elderly aged 65 and over according to individual chronic conditions in the study population

	prev.	mean
Lipid metabolism disorders	28.6	33.37
Hypertension	45.0	33.83
Tobacco abuse	0.9	34.95
Obesity	7.0	35.29
Thyroid diseases	12.8	35.36
Liver disease	7.1	35.47
Purine/pyrimidine metabolism disorders/Gout	11.1	35.50
Hypotension	1.2	35.61
Chronic cholecystitis/Gallstones	4.3	36.62
Lower limb varicosis	10.3	36.63
Noninflammatory gynaecological problems	4.8	37.06
Prostatic hyperplasia	10.0	37.36
Chronic low back pain	26.7	37.63
Hemorrhoids	3.6	37.91
Joint arthrosis	17.2	37.91
Urinary tract calculi	2.1	38.06
Chronic ischemic heart disease	17.6	38.19
Migraine/chronic headache	1.9	38.25
Chronic gastritis/GERD	5.8	38.46
Asthma/COPD	10.1	38.47
Diabetes mellitus	18.8	38.59
Allergy	4.0	38.61
Osteoporosis	6.5	39.46
Sexual dysfunction	1.3	39.48
Severe hearing loss	2.5	39.80
Intestinal diverticulosis	2.9	40.00
Dizziness	2.6	40.11
Psoriasis	1.6	40.25
Severe vision reduction	13.6	40.34
Cardiac arrhythmias	10.9	40.79
Insomnia	3.9	40.79
Somatoform disorders	3.5	40.82
Atherosclerosis/PAOD	8.1	41.14
Cerebral ischemia/Chronic stroke	5.4	41.35
Cardiac insufficiency	6.1	41.57
Depression	7.4	41.70
Rheumatoid arthritis/Chronic polyarthritis	2.3	42.20
Anxiety	1.3	42.41
Cancer	11.6	42.73
Cardiac valve disorders	3.0	42.97
Parkinson's disease	1.3	45.21
Neuropathies	4.6	45.46
Dementia	2.6	45.72
Urinary incontinence	2.7	47.35
Renal insufficiency	3.3	52.00
Anemia	2.0	53.56

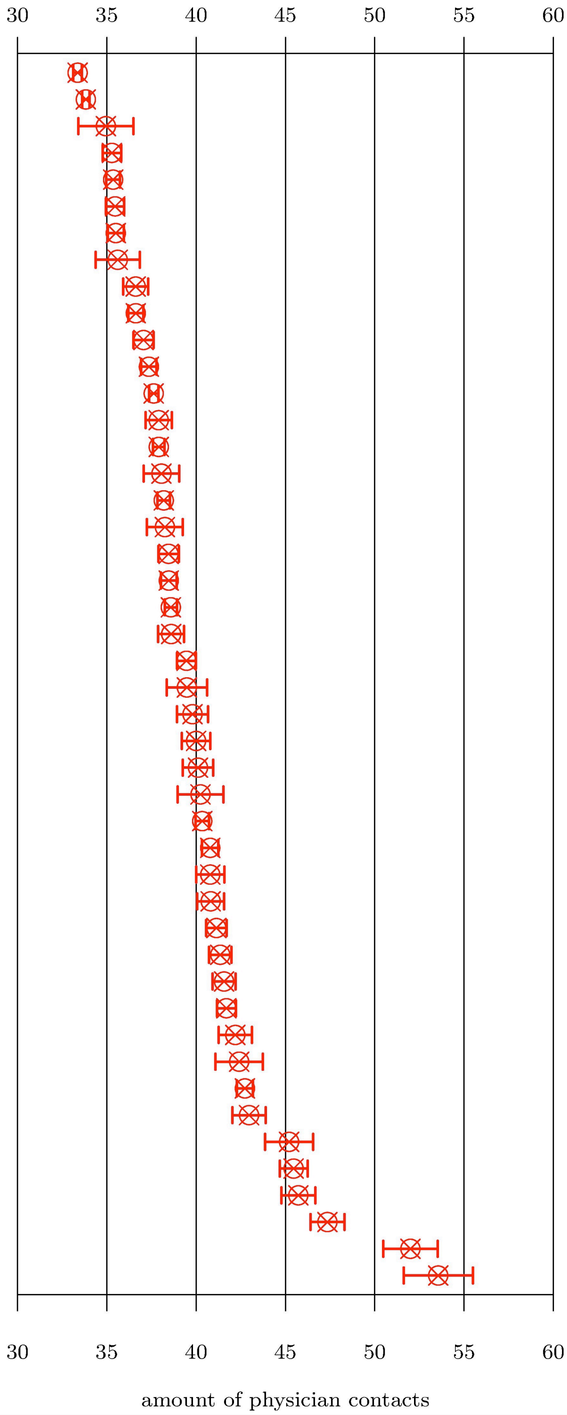

prev = prevalence; red cross in cercle = mean; bars = confidence interval
