# Supplementary material for: Patterns of ambulatory medical care utilization in elderly patients with special reference to chronic diseases and multimorbidity - Results from a claims data based observational study in Germany
Source: BMC Geriatr. 2011 Sep 13;11:54. doi: 10.1186/1471-2318-11-54 (PMC3180370; doi:10.1186/1471-2318-11-54)
Supplement: Additional file 4 — Mean number of contacts per year with physicians in ambulatory care in the multimorbid elderly aged 65 and more according to triadic combinations of chronic conditions ranked according to number of contacts (PDF). [file 1471-2318-11-54-S4.PDF]

Additional file 4: Mean number of contacts per year with physicians in ambulatory care in the multimorbid elderly aged 65 and more according to triadic combinations of chronic conditions ranked according to number of contacts

Nr.	Triadic combination of chronic conditions	Mean	SD	CI
49	hypertension (1) + chronic low back pain (3) + cancers (40)	48.1	27.3	47.1 - 49.1
18	hypertension (1) + chronic low back pain (3) + severe vision reduction (4)	47.4	27.7	46.5 - 48.3
20	hypertension (1) + severe vision reduction (4) + diabetes mellitus (6)	47.1	29.4	46.1 - 48.0
31	hypertension (1) + chronic ischemic heart disease (7) + cardiac arrhythmias (9)	46.5	30.3	45.4 - 47.5
44	chronic low back pain (3) + joint arthrosis (5) + chronic ischemic heart disease (7)	46.1	28.1	45.1 - 47.2
48	chronic low back pain (3) + joint arthrosis (5) + diabetes mellitus (6)	46.1	27.6	45.0 - 47.1
29	hypertension (1) + chronic low back pain (3) + cardiac arrhythmias (9)	45.4	27.5	44.4 - 46.3
32	hypertension (1) + lipid metabolism disorders (2) + cancers (40)	44.9	30.2	43.9 - 46.0
10	hypertension (1) + diabetes mellitus (6) + chronic ischemic heart disease (7)	44.9	29.9	44.0 - 45.7
26	hypertension (1) + joint arthrosis (5) + chronic ischemic heart disease (7)	44.3	28.3	43.4 - 45.2
35	hypertension (1) + chronic low back pain (3) + chronic gastritis/gerd (42)	44.3	27.1	43.3 - 45.3
7	hypertension (1) + chronic low back pain (3) + diabetes mellitus (6)	44.2	27.9	43.4 - 44.9
9	hypertension (1) + chronic low back pain (3) + chronic ischemic heart disease (7)	44.0	27.8	43.2 - 44.7
34	hypertension (1) + chronic low back pain (3) + asthma/copd (16)	43.7	26.8	42.8 - 44.7
17	hypertension (1) + joint arthrosis (5) + diabetes mellitus (6)	43.7	27.4	42.8 - 44.6
27	lipid metabolism disorders (2) + diabetes mellitus (6) + chronic ischemic heart disease (7)	43.4	28.8	42.4 - 44.4
23	lipid metabolism disorders (2) + chronic low back pain (3) + diabetes mellitus (6)	42.8	26.7	41.9 - 43.7
12	hypertension (1) + lipid metabolism disorders (2) + severe vision reduction (4)	42.5	25.3	41.7 - 43.3
46	hypertension (1) + chronic low back pain (3) + prostatic hyperplasia* (12)	42.4	26.4	41.4 - 43.4
38	hypertension (1) + chronic ischemic heart disease (7) + purine/pyrimidine metabolism disorders/gout (11)	42.4	30.6	41.3 - 43.5
47	chronic low back pain (3) + joint arthrosis (5) + lower limb varicosis (13)	42.4	25.4	41.5 - 43.3

13	lipid metabolism disorders (2) + chronic low back pain (3) + chronic ischemic heart disease (7)	42.3	26.4	41.5 - 43.1
25	hypertension (1) + lipid metabolism disorders (2) + atherosclerosis/paod (18)	42.1	30.4	41.1 - 43.1
4	hypertension (1) + chronic low back pain (3) + joint arthrosis (5)	41.8	25.4	41.2 - 42.4
16	hypertension (1) + lipid metabolism disorders (2) + cardiac arrhythmias (9)	41.5	27.0	40.6 - 42.3
21	hypertension (1) + diabetes mellitus (6) + purine/pyrimidine metabolism disorders/gout (11)	41.2	30.0	40.3 - 42.2
43	hypertension (1) + lipid metabolism disorders (2) + chronic gastritis/gerd (42)	41.0	28.0	40.0 - 42.1
19	hypertension (1) + chronic low back pain (3) + lower limb varicosis (13)	41.0	25.4	40.2 - 41.8
15	hypertension (1) + chronic low back pain (3) + thyroid dysfunction (8)	40.8	25.6	40.0 - 41.6
39	hypertension (1) + joint arthrosis (5) + lower limb varicosis (13)	40.8	26.0	39.8 - 41.7
41	lipid metabolism disorders (2) + diabetes mellitus (6) + purine/pyrimidine metabolism disorders/gout (11)	40.7	28.8	39.6 - 41.7
36	hypertension (1) + lipid metabolism disorders (2) + asthma/copd (16)	40.6	26.1	39.6 - 41.5
8	lipid metabolism disorders (2) + chronic low back pain (3) + joint arthrosis (5)	40.5	24.9	39.9 - 41.2
40	hypertension (1) + diabetes mellitus (6) + obesity (10)	40.2	26.7	39.2 - 41.1
50	hypertension (1) + joint arthrosis (5) + purine/pyrimidine metabolism disorders/gout (11)	40.1	25.6	39.1 - 41.0
33	hypertension (1) + lipid metabolism disorders (2) + prostatic hyperplasia* (12)	39.9	26.3	39.0 - 40.9
42	lipid metabolism disorders (2) + chronic low back pain (3) + lower limb varicosis (13)	39.9	24.3	39.0 - 40.8
14	hypertension (1) + chronic low back pain (3) + purine/pyrimidine metabolism disorders/gout (11)	39.9	26.4	39.1 - 40.7
2	hypertension (1) + lipid metabolism disorders (2) + chronic ischemic heart disease (7)	39.6	27.7	38.9 - 40.2
45	hypertension (1) + chronic low back pain (3) + obesity (10)	39.5	25.9	38.6 - 40.5
3	hypertension (1) + lipid metabolism disorders (2) + diabetes mellitus (6)	39.5	27.1	38.8 - 40.1
24	lipid metabolism disorders (2) + chronic low back pain (3) + purine/pyrimidine metabolism disorders/gout (11)	39.1	25.5	38.3 - 40.0
6	hypertension (1) + lipid metabolism disorders (2) + joint arthrosis (5)	39.0	25.1	38.3 - 39.6
37	lipid metabolism disorders (2) + chronic low back pain (3) + thyroid dysfunction (8)	38.8	24.1	37.9 - 39.6
1	hypertension (1) + lipid metabolism disorders (2) + chronic low back pain (3)	38.5	24.5	38.0 - 39.0
22	hypertension (1) + lipid metabolism disorders (2) + lower limb varicosis (13)	37.7	25.2	36.9 - 38.5

5	hypertension (1) + lipid metabolism disorders (2) + purine/pyrimidine metabolism disorders/gout (11)	37.1	27.3	36.4 - 37.8
11	hypertension (1) + lipid metabolism disorders (2) + thyroid dysfunction (8)	36.4	24.5	35.7 - 37.2
28	hypertension (1) + lipid metabolism disorders (2) + obesity (10)	36.1	25.9	35.2 - 37.0
30	hypertension (1) + lipid metabolism disorders (2) + liver disease (14)	36.1	25.0	35.2 - 36.9

* in male subsample only
Mean = mena number of contacts/yeaR
SD = standard deviation
CI = confidence interval
Numbers in row 1 refer to the prevalence rank order of the triad from 1 to 50
Numbers in brackets refer to the prevalence rank order of the individual chronic condition from 1 to 46 [e.g. row nr. 49: hypertension (1) = rank 1 in prevalence].
