# Supplementary material for: Patterns of ambulatory medical care utilization in elderly patients with special reference to chronic diseases and multimorbidity - Results from a claims data based observational study in Germany
Source: BMC Geriatr. 2011 Sep 13;11:54. doi: 10.1186/1471-2318-11-54 (PMC3180370; doi:10.1186/1471-2318-11-54)
Supplement: Additional file 5 — Mean number of physicians contacted per year in ambulatory care in the elderly population according to individual chronic conditions (PDF). [file 1471-2318-11-54-S5.PDF]

Additional file 5: Mean number of physicians contacted per year in ambulatory care in the elderly population according to individual chronic conditions

	prev.	mean
Dementia	2.6	4.99
Cardiac insufficiency	6.1	5.22
Tobacco abuse	0.9	5.28
Hypertension	45.0	5.37
Purine/pyrimidine metabolism disorders/Gout	11.1	5.38
Diabetes mellitus	18.8	5.40
Obesity	7.0	5.43
Cerebral ischemia/Chronic stroke	5.4	5.49
Lipid metabolism disorders	28.6	5.49
Liver disease	7.1	5.56
Chronic ischemic heart disease	17.6	5.70
Chronic cholecystitis/Gallstones	4.3	5.76
Lower limb varicosis	10.3	5.79
Parkinson's disease	1.3	5.80
Psoriasis	1.6	5.90
Cardiac arrhythmias	10.9	5.94
Atherosclerosis/PAOD	8.1	5.97
Thyroid diseases	12.8	5.97
Asthma/COPD	10.1	6.01
Insomnia	3.9	6.03
Urinary incontinence	2.7	6.05
Joint arthrosis	17.2	6.05
Hypotension	1.2	6.06
Renal insufficiency	3.3	6.14
Chronic low back pain	26.7	6.21
Depression	7.4	6.23
Dizziness	2.6	6.24
Osteoporosis	6.5	6.25
Prostatic hyperplasia	10.0	6.26
Urinary tract calculi	2.1	6.32
Anemia	2.0	6.35
Cardiac valve disorders	3.0	6.37
Severe hearing loss	2.5	6.38
Allergy	4.0	6.38
Chronic gastritis/GERD	5.8	6.40
Hemorrhoids	3.6	6.40
Severe vision reduction	13.6	6.41
Migraine/chronic headache	1.9	6.48
Rheumatoid arthritis/Chronic polyarthritis	2.3	6.51
Anxiety	1.3	6.58
Noninflammatory gynaecological problems	4.8	6.66
Intestinal diverticulosis	2.9	6.66
Somatoform disorders	3.5	6.67
Neuropathies	4.6	6.74
Cancer	11.6	6.87
Sexual dysfunction	1.3	6.92

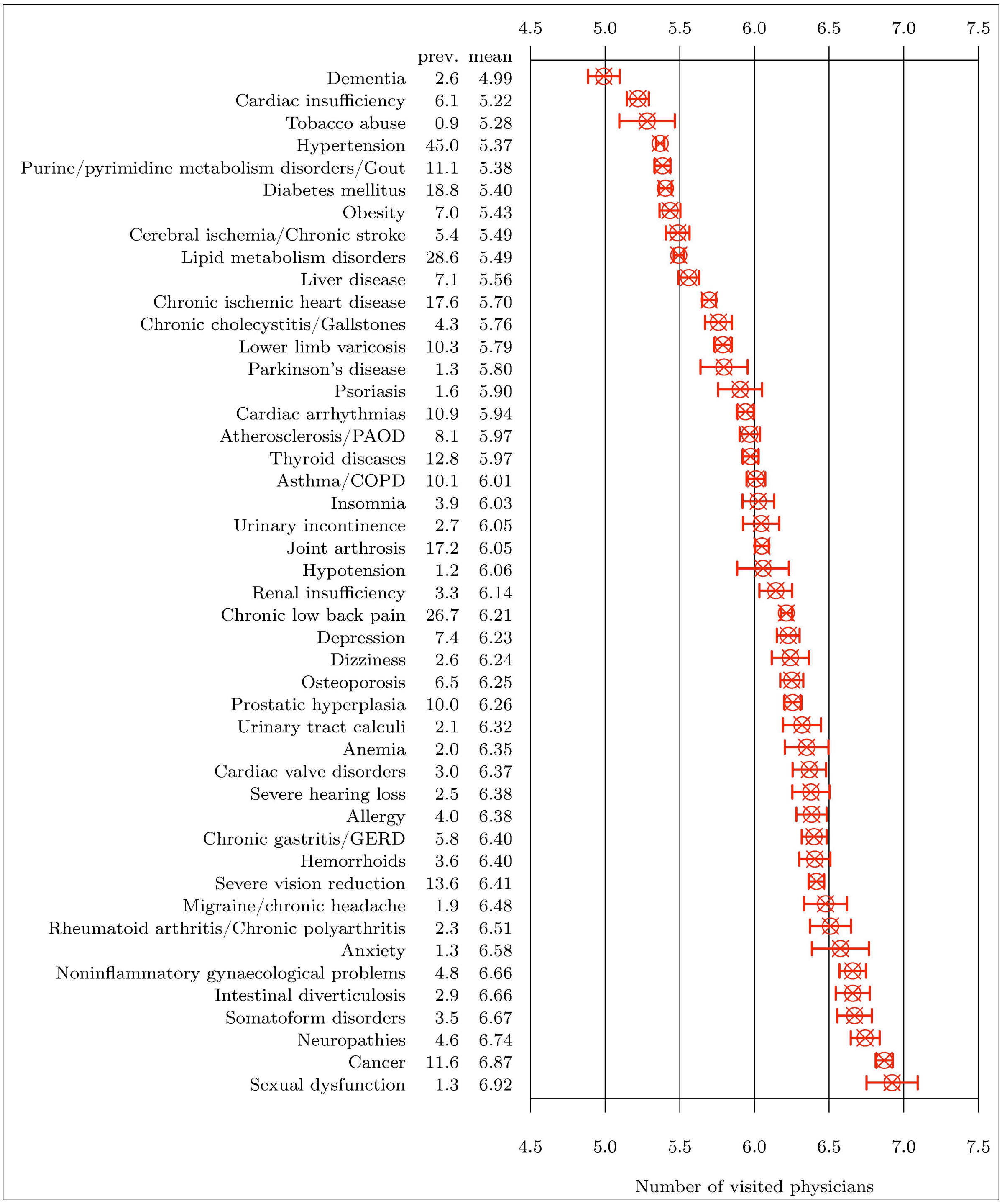

prev = prevalence; red cross in cercle = mean; bars = confidence interval
